# Real-Time Detection of Fouling-Layer with a Non-Intrusive Continuous Sensor (NICS) during Thermal Processing in Food Manufacturing

**DOI:** 10.3390/s21041271

**Published:** 2021-02-10

**Authors:** Fernando José Cantarero Rivera, Dharmendra K Mishra, Ferhan Ozadali, Patnarin Benyathiar

**Affiliations:** 1 Department of Food Science, Purdue University, West Lafayette, IN 47907, USA; fcantare@purdue.edu (F.J.C.R.); fozadali@purdue.edu (F.O.); 2 Reckitt Benckiser Nutrition/Mead Johnson Nutrition, Evansville, IN 47721, USA; 3 Department of Food Technology, Mahidol University, Sai Yok, Kanchanaburi 71150, Thailand; patnarin.ben@mahidol.edu; 4 Islander Consulting and Engineering, Newburgh, IN 47630, USA

**Keywords:** fouling, thermal conductivity, image analysis, composition, thermal resistance

## Abstract

The fouling of indirect shell and coil heat exchanger by heavy whipping cream (HWC) and non-fat dry milk (NFDM) was studied at aseptic Ultra-High Temperature (UHT) processing conditions (140 °C) using a novel non-intrusive sensor. The sensor emitted a heat pulse intermittently throughout the duration of the process causing an incremental increase in temperature at the tube external surface. The temperature response of the sensor varied due to the radial growth of the fouling layer formed by certain components of the products. Each heating pulse and the temperature response was studied to estimate the thermal conductivity of the fouling layer using inverse problems and parameter estimation. The changes in thermal conductivity were used as an indication of the fouling layer development during food processing at UHT temperatures. The estimated parameters from experimental results showed a decreasing trend in the thermal conductivity of HWC and NFDM from 0.35 to 0.10 and 0.63 to 0.37, respectively. An image analysis tool was developed and used to measure the fouling layer thickness at the end of each trial. The measured thickness was found to be 0.58 ± 0.15 for HWC and 0.56 ± 0.07 mm for NFDM. The fouling layer resistance for HWC and NFDM was 5.95 × 10^−3^ ± 1.53 × 10^−3^ and 1.53 × 10^−3^ ± 2.0 × 10^−4^ (m^2^K)/W, respectively.

## 1. Introduction

Fouling is defined as the unwanted accumulation of solids on a surface [1,2,3,4]. In food processing, the most dramatic cases of fouling are typically observed in the dairy industry with the formation of β-lactoglobulin or calcium deposits after milk pasteurization. When dairy products are exposed to high temperatures, proteins unfold, aggregate, and start to deposit at the food contact surface of the system [5]. The deposition depends on the wall temperature but not on the bulk temperature especially in indirect heaters [6]. As production time progresses, the deposit grows over time. The deposit or fouling layer reduces the cross-sectional diameter of the pipe and thereby increases the flow rate, pressure, and thermal resistance [3]. This leads to an increase in the overall heating load of the system to keep the product at the required minimum temperature for product safety from pathogenic microorganisms. An increase in the heating load, therefore, results in higher utility usage, higher power consumption, and increased fuel usage, which cause an environmental impact [7]. About 80% of total production costs in the dairy industry are from fouling and cleaning of the process equipment. [8]. Current studies in processing technologies have reported the Cleaning in Place (CIP) times of around 4 to 6 h per day for the dairy industry due to fouling [9]. This leads to extra maintenance and prevention costs such as over-dimensioned equipment to compensate for the deposit formation and more frequent cleaning [3].

Traditional methods for fouling detection require either expensive equipment or great effort in dismantling the aseptic system that causes prolonged shut-down times. Wallhäußer et al., [10] have identified several types of fouling: precipitation, particulate/sedimentation, corrosion, chemical reaction, solidification, and biofilm formation. Precipitation happens with the crystallization of salts and oxides. An example of this type is dairy fouling type B, especially when calcium deposits form, which occurs approximately at 140 °C. Precipitation fouling is hard and granular in structure and tends to affect heat transfer more than it does to flow rate [6]. Particulate/sedimentation occurs when particles accumulate on surfaces such as colloids or dust. It is classified as type A dairy fouling when the protein from milk deposits on the surface due to denaturation. The composition of the fouling layer varies depending on the product and process types.

Several detection methods have been developed to detect fouling in the food processing system [10]. Pressure drop can be measured between the inlet and outlet of a system, and a larger pressure drop indicates more fouling layer build up. However, it cannot provide any indication of a specific location of the fouling. Another indicator of fouling is the product temperature which can be measured at the product outlet and in the heating medium. The excessive fouling build-up is indicated by a rise in the heating media temperature. Pressure and temperature detection methods, however, lack sensitivity and are unstable at Ultra-High Temperature (UHT) conditions. Other novel methods include measurement of the electrical resistance or acoustic energy dissipation, but these require the use of invasive and cumbersome technology.

Even though comprehensive information of the industrial and laboratory techniques to monitor fouling is available, most of those methods either require expensive instrumentation, transparent equipment, or are lab-scale which is not applicable to the industrial scale systems [11]. The inexpensive thermal methods such as the heat flux sensor also lack accuracy compared to the expensive optical or ultrasonic methods. Currently, there are no sensors that can detect the fouling layer development and thermal properties of the fouling layer. Therefore, the objective of this study was to develop a non-intrusive sensor that can measure the thermal conductivity of the fouling layer formation. A Non-Intrusive Continuous Sensor (NICS) has been developed that can be easily implemented in current commercial systems [12]. NICS not only can detect the buildup of fouling but also can estimate the thermal properties and thermal resistance. It will be able to estimate the fouling thickness as well.

## 2. Materials and Methods

### 2.1. Product Description and Equipment Design

**Figure 1:**
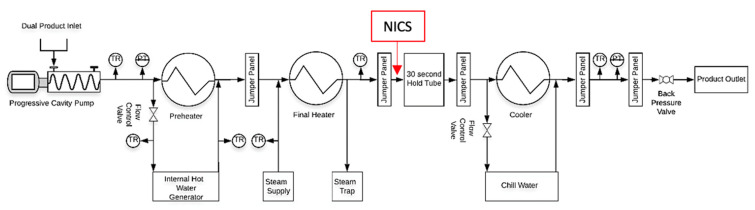
Process flow diagram of the Ultra-High Temperature (UHT) system used for fouling thickness determination.

Grade A Nonfat Dry Milk (NFDM) (Michigan Milk Producers Association, Ovid, MI, USA) reconstituted 15% (*w*/*w*), and Kroger™ Heavy Whipping Cream (HWC) (The Kroger Co. Cincinnati, OH, USA) were selected as the fouling producing agents in the experiments. Their heat capacities and densities for simulation were obtained from the literature [13,14]. All products were processed at a flow rate of 1 L/min with a holding time of 30 s in a MicroThermics^®^ 25 HV lab scale UHT/HTST unit (MicroThermics Inc., Raleigh, NC, USA) to replicate an industrial scale UHT processing system (Figure 1). Temperature in the preheater was set to 90 °C while the final heat exchanger was set to achieve 140 °C at the end of the hold tube.

The embedded thermocouples in the NICS were able to record temperature during the trials. The thermal process was designed to accelerate the fouling in a 1-h trial. NICS was set to pulse every 5 min during the product trial. A single heat pulse experiment including heating and cooling lasted approximately 150 s to allow the system to equilibrate with flowing product temperature before the next experimental pulse. All data was generated and recorded with National Instruments™ (NI, Austin, TX, USA) modules and the sensor was controlled through the custom-made NI LabVIEW software. A more detailed description of the sensor design and calibration process can be found in [12].

### 2.2. Fouling Layer Model

A comprehensive computational fluid dynamics (CFD) model with heat transfer coupled with the fluid flow was developed in COMSOL Multiphysics^®^ 5.5. The model was a 2D axisymmetric representation of a liquid product flowing through a one-inch-long section of a stainless-steel pipe with an outside diameter of 9.525 mm and a tube wall thickness of 0.889 mm. All material properties used for the model are listed in Table 1.

The model included a non-isothermal flow of the liquid product and conjugate heat transfer for the solids in the geometry. The heat transfer equation for fluids and solids was derived using Fourier’s Law and were represented as Equations (1) and (2) , respectively [17]:

(1) $$\rho C\left(\frac{\partial T}{\partial t}+u\cdot \nabla T\right)+\nabla \cdot \left(q+q_{r}\right)=\alpha_{p}T\left(\frac{\partial p}{\partial t}+u\cdot \nabla p\right)+\tau :\nabla u+Q$$

(2) $$\rho C\left(\frac{\partial T}{\partial t}+u_{trans}\cdot \nabla T\right)+\nabla \cdot \left(q+q_{r}\right)=Q_{ted}+Q$$

where *ρ* is the density, *C* is the specific heat capacity, *T* is the absolute temperature, *t* is time, **u** is the velocity magnitude, **u**_trans_ is the velocity vector of translational motion, **q** is the heat flux by conduction, **q**_r_ is the heat flux by radiation, *α_p_* is the coefficient of thermal expansion, *p* is the pressure, *τ* is the viscous stress tensor, *Q_ted_* is thermoelastic damping heat source, **Q** contains additional heat sources.

**Figure 2:**
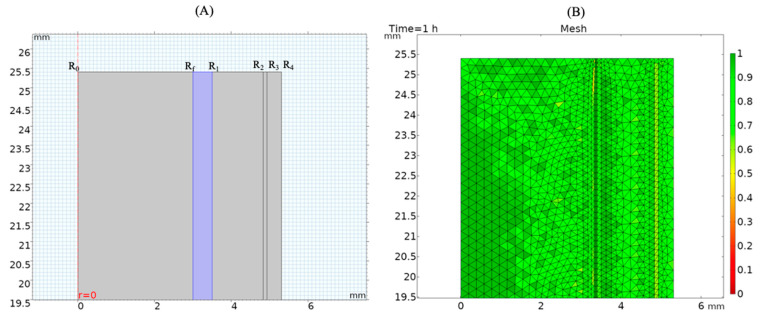
(**A**) Model geometry highlighting different layers: fouling (R_f_–R_1_), product (R_0_–R_f_), stainless steel pipe wall (R_1_–R_2_), air gap (R_2_–R_3_), and sensor (R_3_–R_4_); and (**B**) Model mesh quality (skewness) evaluation.

Since the fluid flow was set to incompressible flow, the thermal conductivity and the heat capacity were evaluated at the default reference temperature of 140 °C. As well, viscous dissipation effects and work done by pressure changes are neglected [17]. Thus, Equations (1) and (2) can be broken down for each specific layer as has been shown by Cantarero et al. [12]:

(3) $$\frac{1}{r}\frac{\partial }{\partial r}\left[k_{h}r\frac{\partial T}{\partial r}\right]+Q=C_{h}\frac{\partial T}{\partial t}\;\mathrm{for}\;R_{3}<r\leq R_{4},t>0$$

(4) $$\frac{1}{r}\frac{\partial }{\partial r}\left[k_{a}r\frac{\partial T}{\partial r}\right]=C_{a}\frac{\partial T}{\partial t}\;\mathrm{for}\;R_{2}<r\leq R_{3},t>0$$

(5) $$\frac{1}{r}\frac{\partial }{\partial r}\left[k_{ss}r\frac{\partial T}{\partial r}\right]=C_{ss}\frac{\partial T}{\partial t}\;\mathrm{for}\;R_{1}<r\leq R_{2},t>0$$

(6) $$\frac{1}{r}\frac{\partial }{\partial r}\left[k_{f}r\frac{\partial T}{\partial r}\right]=C_{f}\frac{\partial T}{\partial t}\;\mathrm{for}\;R_{f}<r\leq R_{1},t>0$$

(7) $$\frac{1}{r}\frac{\partial }{\partial r}\left[k_{p}r\frac{\partial T}{\partial r}\right]=C_{p}\frac{\partial T}{\partial t}\rho Cu\cdot \nabla T\;\mathrm{for}\;R_{0}<r\leq R_{f},t>0$$

where *r* is the thickness of each layer, *k* is the thermal conductivity, and the subscripts *h*, *a*, *ss*, *f* and *p*, represent each of the layers: heater, air, stainless steel, fouling, and product, respectively; *R* represents the different boundaries as shown in Figure 2A.

A predefined mesh size calibrated for fluid dynamics was used to mesh the entire geometry. As shown in Figure 2B, two boundary layers were added at the interface of the product and the fouling layer. Element size ranged from a minimum of 0.00531 mm to a maximum of 0.186 mm. Quality analysis based on skewness, which has a range from 0–1, showed that the average element quality was 0.896.

The initial temperature of product was set as an inflow boundary condition characterized as an inward heat flux:

(8) $$k\nabla T\cdot n=\rho \left(\int_{T_{ustr}}^{T}C\cdot dT\right)u\cdot n$$

where **n** is the normal vector on the boundary, and *T_ustr_* is the upstream temperature which was defined at 140 °C.

The boundary condition at *R*_4_ was set to be a natural convection heat flux condition, expressed as:

(9) $$q_{0}=h\left(T_{ext}-T\right)\mathrm{for}\;R_{4}$$

where *q*_0_ is the boundary convective heat flux, *h* is the heat transfer coefficient, *T_ext_* is the external temperature, and *T* is the temperature at the boundary.

The single-phase flow was based on the Navier-Stokes equations:

(10) $$\rho \frac{\partial u}{\partial t}+\rho (u\cdot \nabla )u=\nabla \cdot [-pl+\tau ]+F$$

(11) $$\frac{\partial \rho }{\partial t}+\nabla \cdot (\rho u)=0$$

where **I** is the identity matrix, *τ* is the viscous stress tensor, and **F** is the volume force vector. Equation (10) represents the conservation of momentum and Equation (11) is the continuity equation and represents conservation of mass. Since incompressible flow was determined, Equation (11) reduces to:

(12) ρ∇⋅u=0

This system contained a no slip wall condition between the product and the pipe wall represented by:

(13) u⋅n=0

Fully developed flow was considered and therefore the flow to the domain was assumed to enter through a straight channel and the tangential flow in the boundary was zero:

(14) u−(u⋅n)n=0

Pressure boundary has been defined to suppress backflow, and is represented by:

(15) $$\left[-pI+\tau \right]n=-{\hat{p}}_{0}n$$

(16) $${\hat{p}}_{0}\leq p_{0}$$

where ^ denotes the average over that boundary [18].

To be able to model this problem more precisely, the mesh was set to a maximum element size for the fouling layer of 0.186 mm. For the rest of the model, it was defined at 0.239 mm. Mesh element quality regarding skewness was analyzed to confirm the convergence of results (Figure 2B). The highest value for skewness was 1 based on the skew that was applied to penalize elements with larger or smaller angles than a perfect element. The average element quality in the model was 0.87 with a single element at a minimum of 0.4661.

### 2.3. Sensor Calibration

The sensor’s controls were calibrated to generate a consistent pulse every 5 min of processing so that the results were not affected in any way due to the previous pulse. Every pulse lasted for 15 s and delivered ~48 W of power. As the pulse would heat up the sample, two thermocouples would read the surface temperature with an uncertainty of ±0.0683 °C. The ability to estimate the thermal conductivity of different products at 140 °C was tested against water, glycerol, a 4% potato starch solution (*w*/*w*), and reconstituted NFDM at 10% (*w*/*w*).

### 2.4. Inverse Problem

In the inverse problem, the thermal conductivity of the fouling layer was estimated as an unknown parameter while all other known properties were defined in the model. The model designed in COMSOL used the heat transfer and fluid dynamics interfaces in a time-dependent study. Literature values were used for the material properties including product and fouling domains. The product was set at an inflow upstream temperature of 140 °C. A natural external convective heat flux boundary was set at the outermost boundary. The parameter must be sensitive to the changes in the measured temperature to estimate the unknown parameter. To understand this effect, the scaled sensitivity coefficients were determined.

The scaled sensitivity of the thermal conductivity was analyzed to ensure proper estimation of the parameter. A sensitivity coefficient is the degree of change in the observed variable (temperature) due to changes in the estimated parameter (thermal conductivity). It is expressed as:

(17) $$X_{k}^{}=\frac{\partial T}{\partial k}$$

where *X_k_* is the sensitivity coefficient of temperature with regards to thermal conductivity [19]. This is often multiplied by the parameter value to create a scaled sensitivity coefficient (SSC) which can be compared easily against the observed data.

(18) $$X_{k}^{\prime }=k\frac{\partial T}{\partial k}$$

In general, the SSC is useful to determine the accuracy of the estimated parameter. A large and uncorrelated SSC is preferred.

Sequential estimation of the parameters was done by using the matrix inversion lemma and Gauss minimization functions. This was done by the following equation [20]:

(19) $$S={\left[Y-\hat{Y}\left(\beta \right)\right]}^{T}W\left[Y-\hat{Y}\left(\beta \right)\right]+{\left[\mu -\beta \right]}^{T}U\left[\mu -\beta \right]$$

where *S* is the sum of squares, *Y* is the experimental response variable, $\hat{Y}$ is the predicted response, *μ* is prior information of parameter *β*, *W* is the inverse of the covariance matrix of errors, *U* is the inverse of covariance matrix of parameters.

The standard error assumptions apply for the sequential estimation of a parameter. This includes that errors have a zero mean, constant variance, normal distribution and are additive and uncorrelated [21]. The iterative sequential procedure from Beck and Arnold, [19] was used:

(20) $$A_{i+1}=P_{i}X_{i+1}^{T}$$

(21) $$\Delta_{I+1}=\phi_{i+1}+X_{i+1}A_{i+1}$$

(22) $$K_{i+1}=A_{i+1}\Delta_{i+1}^{-1}$$

(23) $$e_{i+1}=Y_{i+1}-{\hat{Y}}_{i+1}$$

(24) $$b_{i+1}^{*}=b_{i}^{*}+K_{i+1}\left[e_{i+1}-X_{i+1}\left(b_{i}^{*}-b\right)\right]$$

(25) $$P_{i+1}=P_{i}-K_{i+1}X_{i+1}P_{i}$$

where *A* is the inversion matrix, *P* is the covariance matrix, *X* is the sensitivity matrix, ∆ is the sequential delta, *K* is the gain matrix, *e* is the error vector, *b* is the parameter index, and *i* is the iteration index. This process has the stopping criteria:

(26) $$\frac{\left|b_{j}^{k+1}-b_{j}^{k}\right|}{\left|b_{j}^{k}\right|+\delta_{1}}<\delta$$

### 2.5. Image Analysis

Photos of the fouled tube were taken inside a photo lightbox to ensure uniformity of light in all photos. An interactive interface was created in MATLAB that displayed certain prompts to guide the user for post-processing of the photos. The interface allowed the measurement of different regions of interest (ROI), which was used to measure fouling layer thickness. This data was used in further calculations to estimate the thermal conductivity and to calculate the corresponding thermal resistance of the fouling layer.

## 3. Results and Discussion

### 3.1. Sensor Calibration

Sequential estimation of parameters updates the parameter estimate as new data points are added. The estimate is expected to reach a constant value considered to be the real parameter value [22]. Initial guesses for the thermal conductivity of glycerol, starch, 10% NFDM, and water were 0.3, 0.5, 0.5, and 0.6, respectively. The mean estimated thermal conductivity for starch, NFDM, water, and glycerol and their corresponding mean standard deviations along with their 95% confidence intervals are shown in Table 2. A more detailed account and discussion of the sensor’s accuracy and precision has been discussed in [12].

### 3.2. Fouling Layer Image Analysis

**Figure 3:**
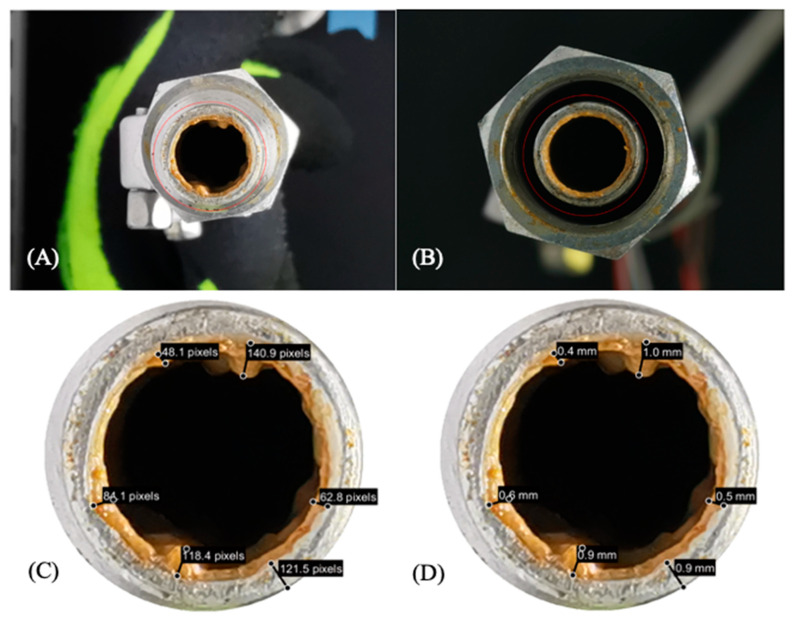
Photo of a fouled tube section mounted with Non-Intrusive Continuous Sensor (NICS) after (**A**) Heavy Whipping Cream (HWC) trial and (**B**) Non-Fat Dry Milk (NFDM) trial. (**C**) Regions of Interest (ROI) lines measuring pixels and scaling factor window, and (**D**) ROI lines measurements converted to corresponding units.

A photo of the fouled tube was analyzed after every processing trial of NFDM and HWC (Figure 3A,B). The original photo was cropped and zoomed in (Figure 3C,D) to be able to measure the thickness of the observed fouling layer.

The code allows for multiple ROI to be drawn as lines and displaying their distances within the image in pixels. Five different sections of the fouling layer were selected to obtain comprehensive data on the fouling layer. Once these selections had been made, the pipe wall was measured and its distance in pixels was used as a reference distance to convert from pixels to millimeters

The mean thickness for the fouling layer created after processing HWC and 15% NFDM was 0.58 ± 0.15, 0.56 ± 0.07 mm, respectively. Figure 3A shows a fouled tube after an HWC trial where there seems to be no symmetry in the formation of the fouling layer. NFDM trials do not show this behavior (Figure 3B). The result of this study also found a similar pattern of fouling layer as reported by Srichantra et al., [23] and Tuoc [6]. The fouling rate of skim milk was found to be higher than the reconstituted and recombined milk (i.e., skim milk + milk fat). Skim milk also forms a thinner fouling layer which looks smooth and glassy, compared to whole milk. This might explain why the fouling layer from processing HWC has no apparent pattern. Based on HWC results, it can be concluded that not all products will form a consistent fouling layer. Therefore, products with less fouling tendency and the non-uniform layer will have a larger error as compared to the products that create more fouling and a uniform layer. A longer processing time might create more consistent fouling layer.

### 3.3. Fouling Layer Thermal Conductivity Estimation

Data at every 5 min in a 1-h long trial was used for every replicate of 15% NFDM and HWC to estimate the thermal conductivity of the fouling layer as time progressed. However, the system would take approximately 10 min to stabilize entirely once the transition from water to the product was made and the recirculation had started.

**Figure 4:**
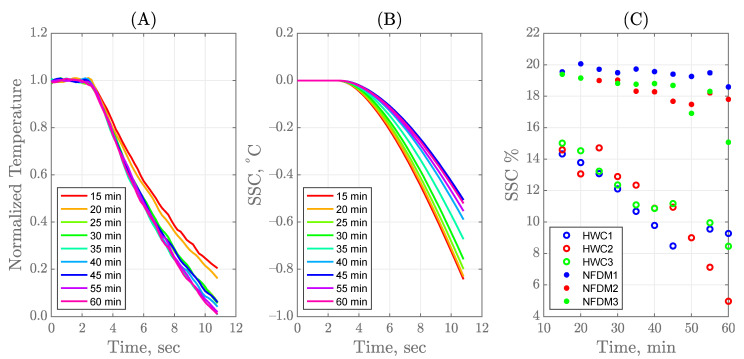
(**A**) Normalized temperature of all experiments for one HWC trial, (**B**) Scaled sensitivity coefficient (SSC) for estimation of the thermal conductivity of HWC at 140 °C, and (**C**) percentage of the change in temperature represented by the SSC at each data point for HWC and NFDM.

The normalized temperature ((T−T_max_)/(T_0_−T_max_)) for each trial were plotted to show the trends in temperature caused by the fouling (Figure 4A). As the trial progressed, the rise in temperature measured by the sensor increased. This is expected as the formation of a fouling layer would have a lower thermal conductivity than that of the product. Therefore, less heat from the sensor would then be able to penetrate, increasing the surface temperature. To account for any discrepancies between the model’s convective heat flux (*h*) and the experimental, different *h* values were estimated depending on the changes in temperature rise during the experiment. For this, intervals were made where each set of experiments would have an estimated *h* used for the estimation of *k*. A clear distinction was made between the experiments from 15 to 35 min and those from 40 to 60 min. These two sections had their *h* estimated and were 546.92 ± 6.19 and 557.98 ± 5.60 W/m^2^K. For 15% NFDM trials, the *h* values were 194.73 ± 3.32 and 230.68 ± 2.70 W/m^2^K. This was to be expected due to the difference in the thermal conductivities for both products.

Thermal conductivity was estimated for each 5-min experimental segment once *h* values had been estimated for each section of the trials. As with the sensor calibration, scaled sensitivity coefficients were examined to determine whether the thermal conductivity would be properly estimated using the experimental data. Figure 4B shows the scaled sensitivity coefficient of the estimation process for the HWC data for each experiment during the processing. The model increase in temperature was 6.23 °C and the SSC reached a maximum magnitude of 0.65 °C meaning it represents a 10.5% of the temperature rise. Since the SSC is above 10%, the thermal conductivity can be properly estimated using the model and response variable.

The SSCs were plotted for the duration of the trials. A downward trend can be observed in the percentage of the change in SSC (Figure 4C). This trend is similar to that shown by the value of the thermal conductivity, which was estimated at every 5 min interval for each product. This could imply that the sensitivity of the thermal conductivity of the fouling layer decreases with the increase in sensor temperature. This may be due to the parameter being more dependent on the thickness of the fouling layer instead of the generated heat pulse.

**Figure 5:**
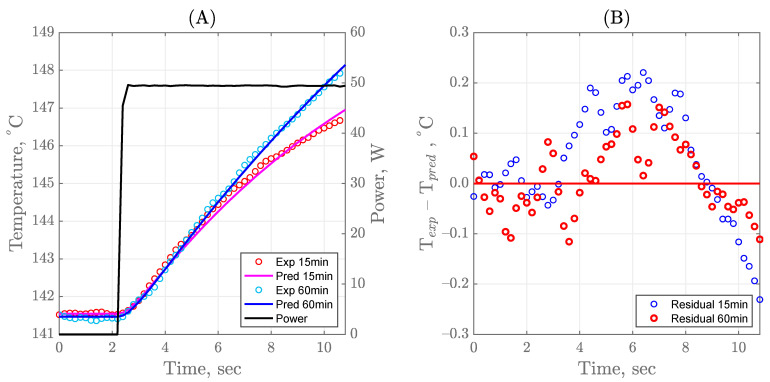
Temperature profile, (**A**) and residual plot, (**B**) for the sensor heat pulse at 15 and 60 min of processing HWC.

To further understand the effect of time on the temperature recorded by the sensor, experimental and predicted data at 15 and 60 min of processing have been plotted to observe any differences between the beginning and the end of the trial (Figure 5A). The model clearly fits the later stage of the process better than it does in the beginning. This difference is made even clearer when the residuals are observed (Figure 5B). A clear trend in the residuals can be observed for the trial at 15 min while the residuals can be considered to have a normal distribution at 60 min. This difference may occur because the fouling layer has a preset thickness in the model. Thus, when it was paired with the initial thermal conductivity, it can cause the model to not be able to heat up as fast as the experiment does. As well, the undershooting of the model at the last 1–2 s may also be due to a preset initial outward convective heat flux value of 400 W/m^2^K when the final estimates showed the real value to be more in the vicinity of ~500 W/m^2^K. This higher value would mean a faster removal of heat from the sensor into the ambient which would result in the observed experimental curve.

**Figure 6:**
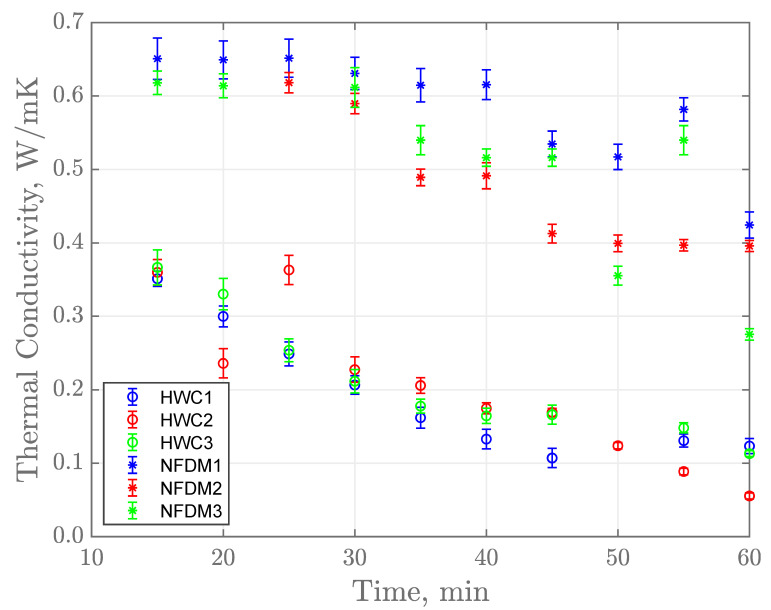
Estimated k average with 95% confidence interval for NFDM and HWC trials.

The average thermal conductivity of NFDM and HWC is shown in Figure 6. Experimental temperatures are plotted starting at 15 min because it takes the system approximately 10 min to stabilize from water to product transition. Thermal conductivity estimates for the fouling layer at the beginning of the trials have a value, which is similar to the original product flowing through the system since there was no fouling at the start of the experiment. HWC thermal conductivity at 15 min was found to be 0.3511 W/(m·K) which is similar to other reported values of cream in the literature 0.42 W/(m·K) [24], 0.33 W/(m·K) [14], and 0.33 W/(m·K) [13]. Thermal conductivity of NFDM at 15 min was 0.6508 W/(m·K) which was also similar to that found in literature, 0.63 W/(m·K) [13].

As observed in Figure 6, the errors are increasing as experimental time progresses. It means that the SSCs for both products are lower with an increase in the fouling on the internal surface which explains why the estimates have larger errors towards the end of the experiment. As well, the model used in this study has a preset thickness for the fouling layer of 0.5 mm whereas experimentally, a thicker layer was observed. This would cause the larger uncertainty around the estimates when more fouling builds up. Hence, this acts as an indication that the system should be cleaned before further production.

A clear downward trend was noticed for the evolution of thermal conductivity as process time progresses (Figure 6). This can be attributable to the formation of the fouling layer. Kazi et al. [25] demonstrated this by proving how prolonged processing times reduced the overall heat transfer coefficient drastically because of fouling formation. Their study comprehended a much longer processing time, however, their process was single pass and the experiments in current study were kept shorter by recirculating the product. Literature shows that fouling formation tends to have an asymptotic growth eventually reaching a stable value where both deposition and resuspension rates balance each other out [6,26]. This might not be as apparent in the NFDM trials but could explain the behavior of the HWC trials. Perhaps the NFDM fouling layer would require a longer time to reach this equilibrium.

### 3.4. Fouling Layer Thermal Resistance Calculations

To calculate the thermal resistance of the fouling layer at the end of the trial, the thickness of the different sections of fouling were paired with the estimated thermal conductivity at the 60-min mark of the process for both products. Thermal resistance was calculated by:

(27) $$R_{f}=\frac{x_{f}}{k_{f}}$$

where *R_f_* is the thermal resistance of the fouling layer in (m^2^·K)/W, *x_f_* is the thickness of the fouling layer evaluated at every ROI in meters and *k_f_* is the estimated thermal conductivity of the fouling layer in W/(m·K). The mean thermal resistance for the fouling layer created after processing HWC and 15% NFDM was 5.95 × 10^−3^ ± 1.53 × 10^−3^ and 1.53 × 10^−3^ ± 2.0 × 10^−4^ (m^2^K)/W, respectively.

The larger standard deviation in the HWC thickness and thermal resistance further demonstrates how the formation of fouling is more random in this than in the NFDM trials. Bouvier et al. [27] and Davies et al. [28] studied the thermal resistance of whey protein deposits by using heat flux sensors and reported values close to 1.0 × 10^−3^ (m^2^·K)/W at about half the processing time used in the current study. None of the previously mentioned studies were able to provide an estimated fouling layer thickness but rather calculated it based on a series of assumptions.

## 4. Conclusions

A non-intrusive sensor, NICS was designed and developed for the determination of fouling in the food manufacturing systems. The non-intrusive nature of the NICS allows it to be quickly and easily adapted to commercial manufacturing systems without any disruption in production. Thermal conductivity of the fouling layer was estimated based on the inverse problems and parameter estimation. NICS was able to detect the fouling in continuous flow system using thermal conductivity as an indicator of fouling. Thermal conductivity of the fouling layers formed by HWC and 15% NFDM under aseptic processing temperatures (140 °C) over a continuous run of 60 min were found to reduce by 39% and 72%, respectively. An image analysis tool was used to aid in measuring the fouling thickness at the end of the process and then use the thickness values to calculate the thermal resistance of the fouling layer. The thickness of the fouling layer at the end of the 60 min trial was 0.58 ± 0.15, 0.56 ± 0.07 mm for HWC and 15% NFDM, respectively. The results also showed that the fouling layer varied in uniformity depending on the product types. NFDM formed a uniform layer as compared to the HWC. The sensor NICS can be easily mounted at several locations in the system to monitor real time fouling layer development. The model can be used in future work to analyze the effect of changes in layer size and layer growth parameters on thermal properties and processing parameters.

## Figures and Tables

**Table 1:** Material properties used in the Computational Fluid Dynamics (CFD) model.

Product/Material	Density (kg/m^3^)	Specific Heat (kJ/kg·K)	Thermal Conductivity (W/m·K)	Reference
Non-Fat Dry Milk (NFDM)	1035	3.94	0.53	[13,14]
Heavy Whipping Cream (HWC)	1001	3.56	0.33	[13,14]
316L Stainless Steel	8000	500.00	16.30	[15]
Non-Intrusive Continuous Sensor (NICS)	3110	862.90	37.30	[16]

**Table 2:** Mean estimated thermal conductivities for all products and their corresponding 95% confidence interval (CI).

Product	Mean Thermal Conductivity (W/(m·K))	Std. Error	Lower CI	Upper CI
Glycerol	0.2919	0.0064	0.2887	0.2952
Water	0.6384	0.0125	0.6329	0.6439
4% Starch	0.4873	0.0086	0.4835	0.4911
10% NFDM	0.5977	0.0104	0.5934	0.6020

## Data Availability

Research data presented in this article are available on request from the corresponding author.
